# Mode-Selective and Anharmonicity-Dependent Energy Transport in Cavity-Coupled Water

**DOI:** 10.1021/acs.nanolett.6c04125

**Published:** 2026-10-09

**Authors:** Sachith Wickramasinghe, Michael Fowler, Gerrit Groenhof, Arkajit Mandal

**Affiliations:** † Department of Chemistry, 14736 Texas A&M University, College Station, Texas 77843, United States; ‡ Department of Chemistry, University of Jyväskylä, 40014 Jyväskylä, Finland

**Keywords:** Vibropolaritons, Vibrational Energy Transport, Light−Matter Interactions, Molecular Cavity Quantum Electrodynamics

## Abstract

Recent experiments demonstrate the modification of chemical dynamics via cavity-enhanced vibrational energy transport. Here, we provide a microscopic account of both photonic and mode-selective energy transport using direct mesoscale on-the-fly simulations of cavity-modified transport under vibrational strong coupling. We find that molecular anharmonicity leads to nonlinear driving-dependent photonic transport and localization, which we demonstrate in cavity-coupled water by tuning the photon frequency close to either the harmonic bending or the anharmonic stretching modes. We reproduce these effects in simple model systems, suggesting that the underlying mechanism extends beyond the specific case of cavity-coupled water. We also demonstrate that the diffusion of mode-selective temperature, quantified via the variance of the H–O–H bond angle or O–H bond length, depends on the cavity frequency, which we rationalize using an analytical model. Our results highlight the rich dynamical interplay of molecular and photonic degrees of freedom that persist in real atomistic systems.

## Graphical abstract

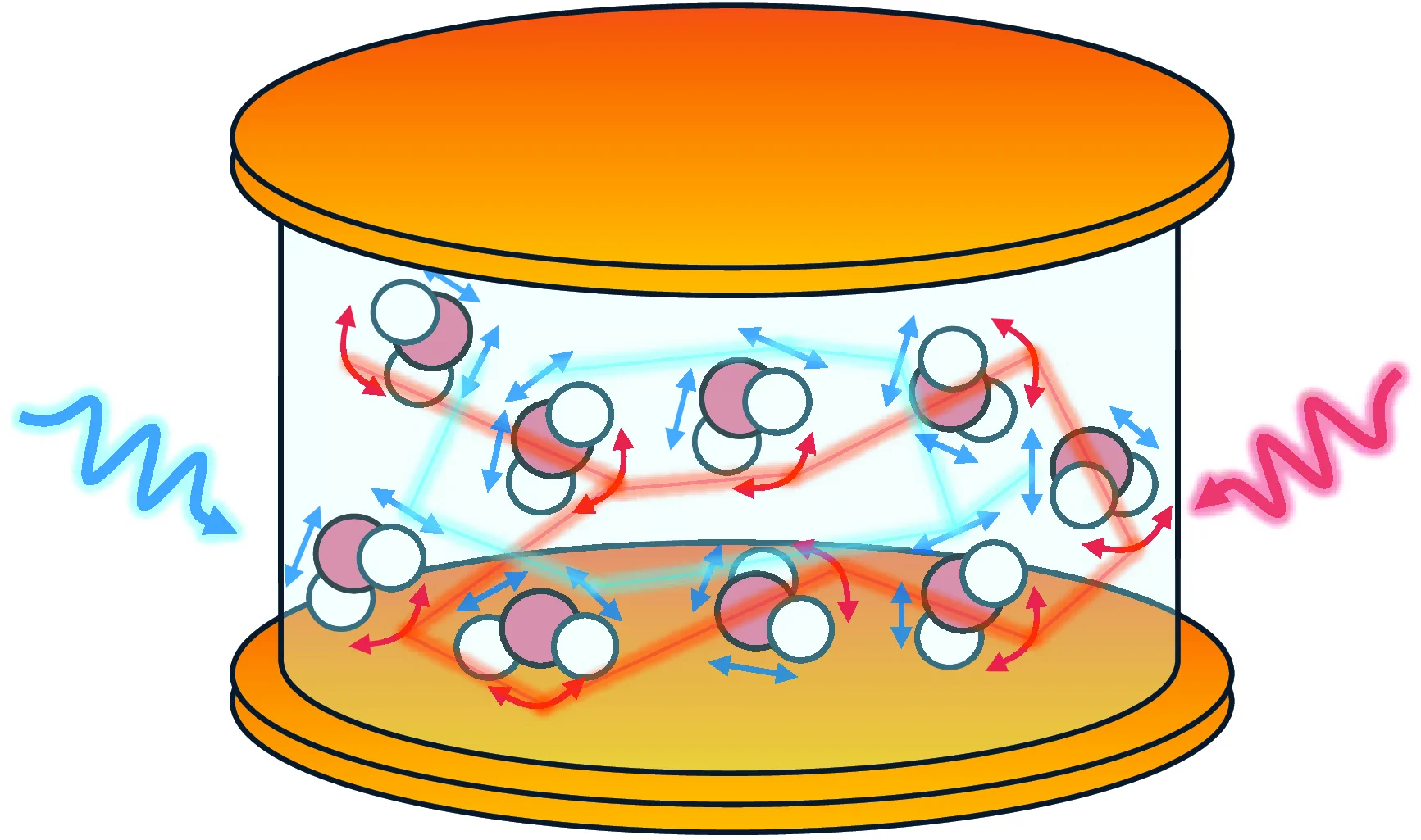

Chemists have long regarded mode-specific control of reactions as a holy grail of chemistry.^1–5^ While synthetic strategies for achieving bond-selective reactions have been developed for decades and are continually being refined, attempts to realize the same goal via selective vibrational excitation with intense laser fields have failed to materialize as a generalized tool for chemical synthesis.^2,6^ This is because any targeted vibrational energy rapidly leaks into other modes via intramolecular vibrational energy redistribution (IVR), which competes with the desired chemical pathway relating to the targeted vibrational excitation.^6,7^ This rapid redistribution arises from anharmonic coupling among the molecule’s normal modes, which enables fast vibrational energy flow through molecular bonds. In contrast, through-space vibrational energy transfer (between two molecules) is significantly slower and rarely plays a dominating role in determining chemical dynamics as it is mediated by substantially weaker intermolecular interactions.^8^

Recent experiments, however, suggest a fundamentally different route: modification of ground-state chemical reactivity via the formation of vibropolaritons^1,4,9–18^ (light–matter hybrid quasi-particles) inside infrared optical cavities in the vibrational-strong-coupling (VSC) regime, where an ensemble of molecular vibrations is coupled to confined radiation fields. Specifically, recent experiments show that a driven VSC system, which is the focus of the present study, enables rapid through-space transport of vibrational excitations between spatially separated molecules over otherwise inaccessible distances and time scales, which has been suggested to modify chemical dynamics.^1,8^ Interestingly, experiments also suggest the modification of chemical kinetics at thermal equilibrium^4,9,10,19–21^ even in the absence of external driving,^22^ albeit with some concerns about these claims due to subtleties in the interpretation of spectroscopic data.^23–26^ Nevertheless, cavity-enabled through-space transport has the potential to unlock the long-sought ability to perform mode-selective chemistry non-invasively and, when fully realized, could represent a paradigm shift in how we think about chemical synthesis. Importantly, the microscopic mechanistic principles of VSC-enabled transport and consequent cavity-modified chemical dynamics remain poorly understood.

In this work, using direct on-the-fly mesoscale simulations, we provide a microscopic account of cavity-modified energy transport in water and establish fundamental mechanistic principles governing photonic transport and mode-selective, through-space vibrational energy transport inside an optical cavity. Specifically, we demonstrate how molecular anharmonicity controls photonic transport and how cavity photon frequency can be tuned to achieve mode-selective energy transport. We find that molecular anharmonicity modulates localization of photonic density, leading to a nonlinear dependence on the driving laser power. We show that when the cavity photon frequency at normal incidence is resonant with nearly harmonic molecular modes, such as the bending mode of water, which exhibits relatively weak anharmonicity, the photonic localization initially increases with pump power before plateauing at higher driving strengths. In contrast, when the cavity is resonant with the significantly more anharmonic water stretching modes, photonic localization exhibits a non-monotonic dependence on pump power. This mirrors our observation in many-body quantum dynamics of exciton–polaritons where the many-body excitonic interactions play a similar role as molecular anharmonicity.^27^ We further demonstrate that tuning the cavity photon frequency can selectively enhance vibrational energy diffusion through either the bending or stretching modes. Together, these results establish mechanistic principles for understanding how molecular anharmonicity and cavity frequency dictate energy transport under VSC in realistic molecular systems.

While not the direct focus of this work, our work also sheds light on a fundamental conundrum of cavity modification of chemical reactivity:^16,28,29^ how can collective cavity couplings, which connect cavity radiation to molecular degrees of freedom in a delocalized manner, with each individual molecule experiencing only a negligible fraction of the total coupling, nevertheless lead to modifications of chemical reactivity that operate locally? The cavity-enhanced energy transport investigated here operates in the collective-coupling regime. By enabling long-range, frequency-selective vibrational energy transport, the cavity provides a mechanism for translating a collective light–matter interaction into a cavity-controlled local modification of chemical reactivity. Such control directly addresses a central bottleneck in mode-selective chemistry: determining where vibrational energy flows on ultrafast time scales. The present work lays the foundation for future investigations into how cavity-modified energy diffusion can be harnessed to control chemical dynamics as well as potential quantum dynamical effects^30–34^ in VSC.

In this work, we perform mesoscale simulations using our recently developed cavOTF framework,^35^ which is formulated in the dipole gauge beyond the long-wavelength approximation.^35–39^ Specifically, we adapt a mixed representation (where parts of the Hamiltonian are represented in reciprocal space while the rest is represented in real space) of the light–matter Hamiltonian, written as^35^

1 $${Ĥ}_{\mathrm{LM}}={Ĥ}_{M}+\underset{k}{\sum }\omega_{k}{â}_{k}^{†}{â}_{k}+\eta \underset{n}{\sum }{\mathrm{q̂}}_{n}{\mathrm{\mu ̂}}_{n}+\frac{\eta^{2}}{2{\omega_{0}}^{2}}\underset{n}{\sum }{{\mathrm{\mu ̂}}_{n}}^{2}$$

Here, ${Ĥ}_{M}$ represents the molecular Hamiltonian which is written as a sum of *N* non-interacting unit cells, following prior works,^35,38,39^ such that

2 $${Ĥ}_{M}=\underset{n}{\sum }{Ĥ}_{M_{n}}=\underset{n,j}{\sum }{\mathrm{T̂}}_{n,j}+{\mathrm{V̂}}_{n}\left(\left\{r_{n,j}\right\}\right)$$

where ${\mathrm{T̂}}_{n,j}$ is the kinetic energy operator of the *j*th atom in the *n*th unit cell, and ${\mathrm{V̂}}_{n}\left(\left\{r_{n,j}\right\}\right)$ is the corresponding potential energy operator. In eq 1, the operators ${â}_{k}^{†}$ $\left({â}_{k}\right)$ denote the photon creation (annihilation) operators corresponding to the *k*th cavity mode with frequency $\omega_{k}=c\sqrt{{\left(\pi /L\right)}^{2}+k^{2}}$ , where $k=\frac{2\pi j}{L_{s}}$ (*j* = 0, ±1, ±2, ...) is the in-plane wavevector with *L*_s_ as the length of the supercell, and *c* is the speed of light. The parameter $\eta =\sqrt{{\omega_{0}}^{3}}g_{0}$ (with *g*_0_ = 1.8936 × 10^–5^ au as the normalized coupling) denotes the collective light–matter coupling strength which has been set to place the system in the vibrational strong coupling regime, ${\mathrm{q̂}}_{n}=\frac{1}{\sqrt{2\omega_{0}}}\left({â}_{n}^{†}+{â}_{n}\right)$ is the real space photonic position operator, ω_0_ is the cavity photon frequency at normal incidence, and ${\mathrm{\mu ̂}}_{n}$ is the total dipole operator of the *n*th unit cell. We propagate the dynamics of the nuclear and photonic degrees of freedom classically, i.e., $\left\{{\mathrm{r̂}}_{n,j},{\mathrm{q̂}}_{n}\right\}\to \left\{r_{n,j},q_{n}\right\}$ . In our approach, we exploit the sparsity of the mixed real-reciprocal space description of the light–matter interaction, devising a low-communication hub-and-spoke server-client parallelization scheme to perform our mesoscale simulations.^35^ The equations of motion for both the nuclear and photonic coordinates are derived from Hamilton’s equations of motion and integrated in time using a velocity-verlet-like scheme where we switch between real and reciprocal space in each nuclear time step. Details of our propagation scheme can be found in ref 35. In addition to ${Ĥ}_{\mathrm{LM}}$ in eq 1, we include a time-dependent laser that drives the photonic modes, such that the total Hamiltonian, ${Ĥ}_{\mathrm{total}}$ , is written as

3 $${Ĥ}_{\mathrm{total}}={Ĥ}_{\mathrm{LM}}+E\left(t\right)\underset{n}{\sum }g_{n}{\mathrm{q̂}}_{n}$$

Here, $g_{n}=g_{L}\underset{m\in W}{\sum }\delta_{nm}$ , with *g*_L_ as the laser driving strength, and *W* defines the spatial window over which the laser is focused, as illustrated in Figure 1a. The temporal profile of the external field is given by $E\left(t\right)=\Theta \left(t\right)\left[1-\Theta \left(t-t_{s}\right)\right]\sin\left(\omega_{l}t\right)$ , where Θ­(*t*) is a Heaviside function, such that the laser is turned on between *t* ∈ [0, *t*_s_]. We set ω_L_ = ω_0_ and *t*_s_ = 10 fs; the short pulse duration produces a broad excitation spectrum.

**Figure 1:**
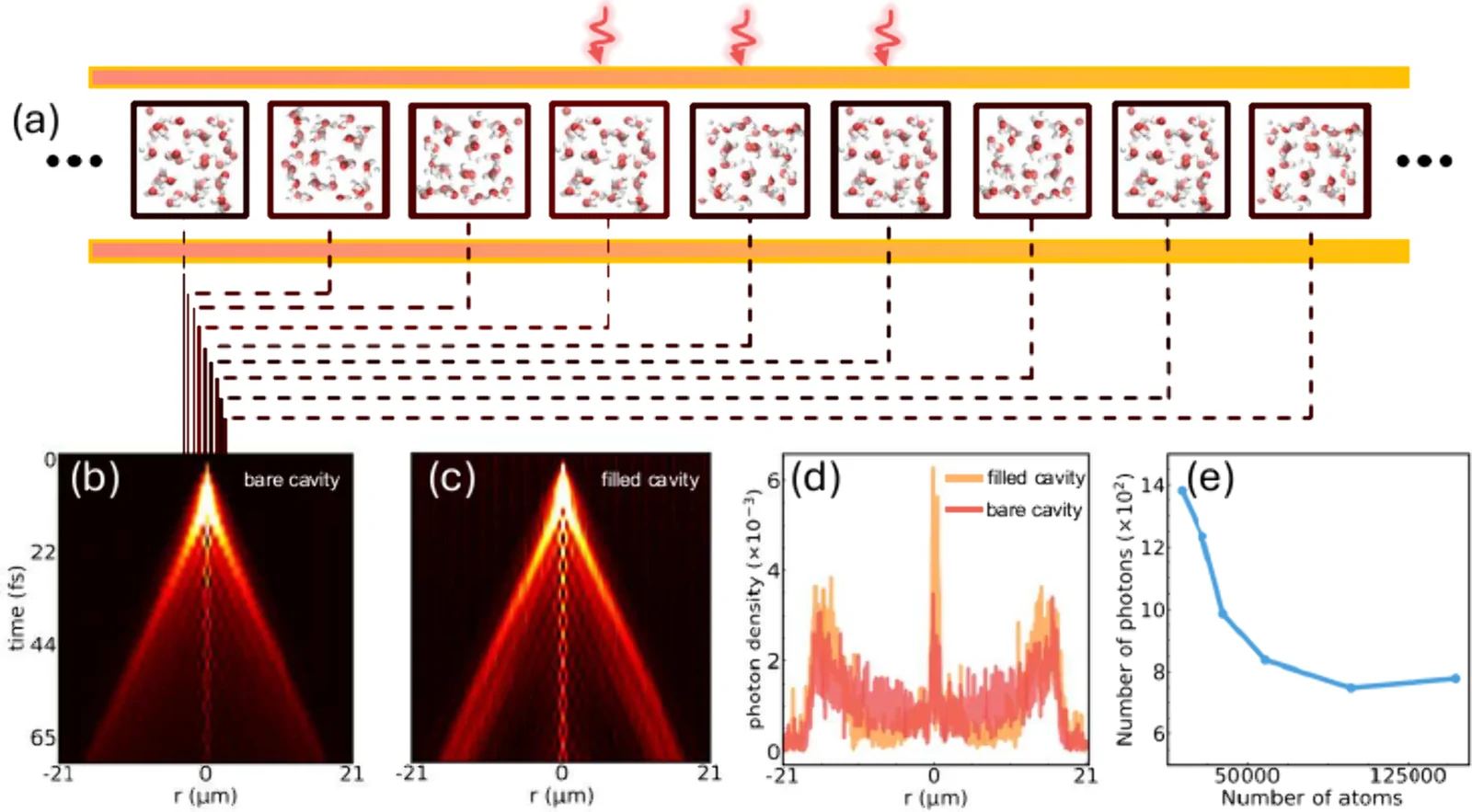
Mesoscale on-the-fly simulations of cavity-coupled water and their convergence. (a) Schematic representation of *N* unit cells of water molecules aligned in an optical cavity with laser-driven central cells. (b) Propagation of photons over 72 fs within a bare cavity. (c) Propagation of photons over 72 fs within a cavity filled with coupled water molecules. (d) Photon density at 72 fs for the filled cavity (orange) and bare cavity (red). (e) Photon number convergence with system size. We set ω_0_ = 0.16 eV and *g*_L_ = 0.6 au.

In our simulation setup, we consider an optical cavity with a total length of *L*_s_ = 8 × 10^5^ (au). We place *N* equally spaced unit cells along the in-plane direction of the cavity. Each unit cell is a periodic cubic cell with a size of 10 Å and contains 33 water molecules, matching the density of bulk water at room temperature.^40^ We use DFTB+ to solve the electronic structure and compute the atomic forces, dipoles, and (numerical) dipole gradients on-the-fly.^35,41,42^ While the present work is focused on the ground state dynamics, our approach could be extended to study excited state VSC utilizing the TD-DFTB approach.^41,43,44^ Initial positions and velocities of the water molecules were sampled from a canonical (*NVT*) molecular dynamics simulation. We used the “SupercellFolding” scheme^45^ to perform electronic structure calculations with SCC-DFTB on a 3 × 3 × 3 folded supercell, where we used Γ-point sampling to approximate Brillouin zone integration. Due to the mesoscale nature of the system, we find that even just one trajectory produces converged results for the types of quantities investigated in the present work. In the following, we use two trajectories to obtain converged results for our mesoscale on-the-fly simulations, with additional results for up to four trajectories presented in the Supporting Information.

## Mesoscale On-the-Fly Simulations of Cavity-Coupled Water

In Figure 1, we present typical mesoscale simulations of vibrational polaritons formed in cavity-coupled water. Our setup is schematically illustrated in Figure 1a. We set *W*, the excitation window, to 11 central unit cells. In Figure 1b, we present the spatially resolved photonic transport in an empty cavity and compare it with the photonic transport in a filled cavity (i.e., cavity-coupled water), shown in Figure 1c, when setting ω_0_ = 0.16 eV. We compute the spatially localized photon number at the *n*th unit cell classically as

4 $$N_{n}\left(t\right)=\frac{1}{2}\left(\frac{{p_{n}}^{2}\left(t\right)}{\omega_{0}}+\omega_{0}{\left(q_{n}\left(t\right)+\frac{\alpha \mu_{n}\left(t\right)}{{\omega_{0}}^{2}}\right)}^{2}\right)$$

where *p*_*n*_ is the photonic momentum (which relates to the vector potential), *q*_*n*_ is the photonic position (which relates to the electric field), ω_0_ is the cavity frequency, and α is a gauge parameter^46^ found by variationally minimizing the total photon number (which is done once for each set of parameters). We note that the choice of α does not alter the overall profile of the spatially resolved photon number presented here.

Overall, here we observe both localized and ballistically propagating photonic density. This is because our broad laser excitation near the bottom of the photonic dispersion creates photonic density with both zero and non-zero group velocities. Coupling to water does change the time-dependent photonic density, as shown in Figure 1d, in that a portion of the photonic density between the fast-propagating and fully localized parts is suppressed. This is due to the fact that the bending mode of water at ω_θ_ = 0.19 eV couples to the photonic density with intermediate group velocity.

Importantly, we find that the coupled molecular-photonic dynamics is sensitive to the number of unit cells *N* considered in the simulation, even though the light–matter coupling is appropriately normalized. To ensure convergence, we have computed multiple observables of interest, one of which, the total number of photons at 72 fs at progressively higher number of unit cells (consequently higher number of total atoms, while keeping the number of atoms per unit cell constant), is presented in Figure 1e. We find that ∼10^5^ atoms are needed to obtain reasonably converged results for the present setup, which we therefore use in our simulations.

## Anharmonicity-Controlled Transport

Experimental results suggest that localization of polaritonic wave functions disrupts vibrational energy transport through the cavity.^1^ However, the present theoretical understanding of this phenomenon^47^ is limited to a single cavity mode model (employing the long-wavelength approximation), which cannot capture polaritonic transport, and uses a simple two-level molecular model that cannot capture rich molecular complexities. Using our on-the-fly simulation, we provide a microscopic account of how energy transport, quantified here via photonic transport, depends on molecular anharmonicity.

To elucidate the role of molecular anharmonicity in photonic transport, we consider two setups; we use a cavity with ω_0_ = 0.16 eV (Figure 2a1–d1) near the bending mode at ω_θ_ ∼ 0.19 eV, which is primarily harmonic in nature, and ω_0_ = 0.40 eV (Figure 2a2–d2) near the stretching modes at ω_*v*1_ ∼ 0.43 eV and ω_*v*2_ ∼ 0.47 eV, which are significantly more anharmonic in nature. The uncoupled dispersions for these two setups are illustrated in Figure 2a1 and a2.

**Figure 2:**
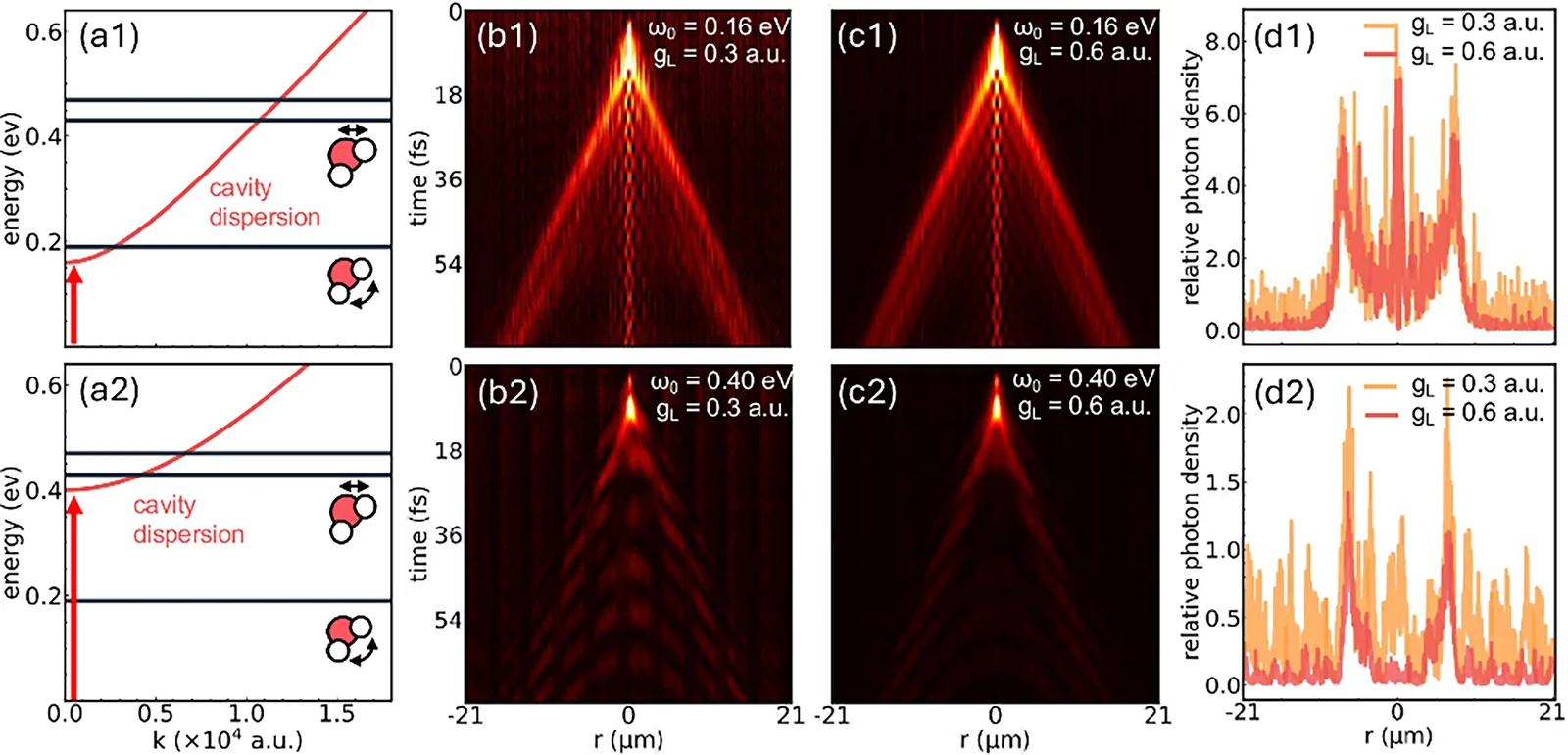
Anharmonicity-controlled transport. (a) Dispersion of cavity (red) when excited at (a1) ω_0_ = 0.16 eV and (a2) ω_0_ = 0.40 eV and energies of water (black) bending (ω_θ_ ∼ 0.19 eV) and stretching (ω_*v*1_ ∼ 0.43 eV and ω_*v*2_ ∼ 0.47 eV) modes. (b) Photonic propagation with driving strength of *g*_L_ = 0.3 au when the cavity frequency is near resonance with (b1) the bending mode and (b2) the stretching modes. (c) Photonic propagation with driving strength of *g*_L_ = 0.6 au when the cavity frequency is near resonance with (c1) the bending mode and (c2) the stretching modes. (d) Relative photon density at 72 fs at driving strengths *g*_L_ = 0.3 au (orange) and *g*_L_ = 0.6 au (red) when the cavity frequency is near resonance with (d1) the bending mode and (d2) the stretching modes.

In Figure 2b1 and c1, we present the spatially resolved time-dependent photonic density at two different driving strengths *g*_L_. Overall, we find that photonic transport is nearly identical when changing *g*_L_ for ω_0_ = 0.16 eV, even though the total number of photons in the cavity setup scales as ${g_{L}}^{2}$ , as expected. This can be further observed in Figure 2d1, which presents the spatially resolved relative photonic density, $N_{n}/{g_{L}}^{2}$ , at 72 fs. The same result is also obtained when using simple harmonic models (as shown in Figure 3); the photonic propagation is independent of the driving strength (beyond a thermal noise floor).

**Figure 3:**
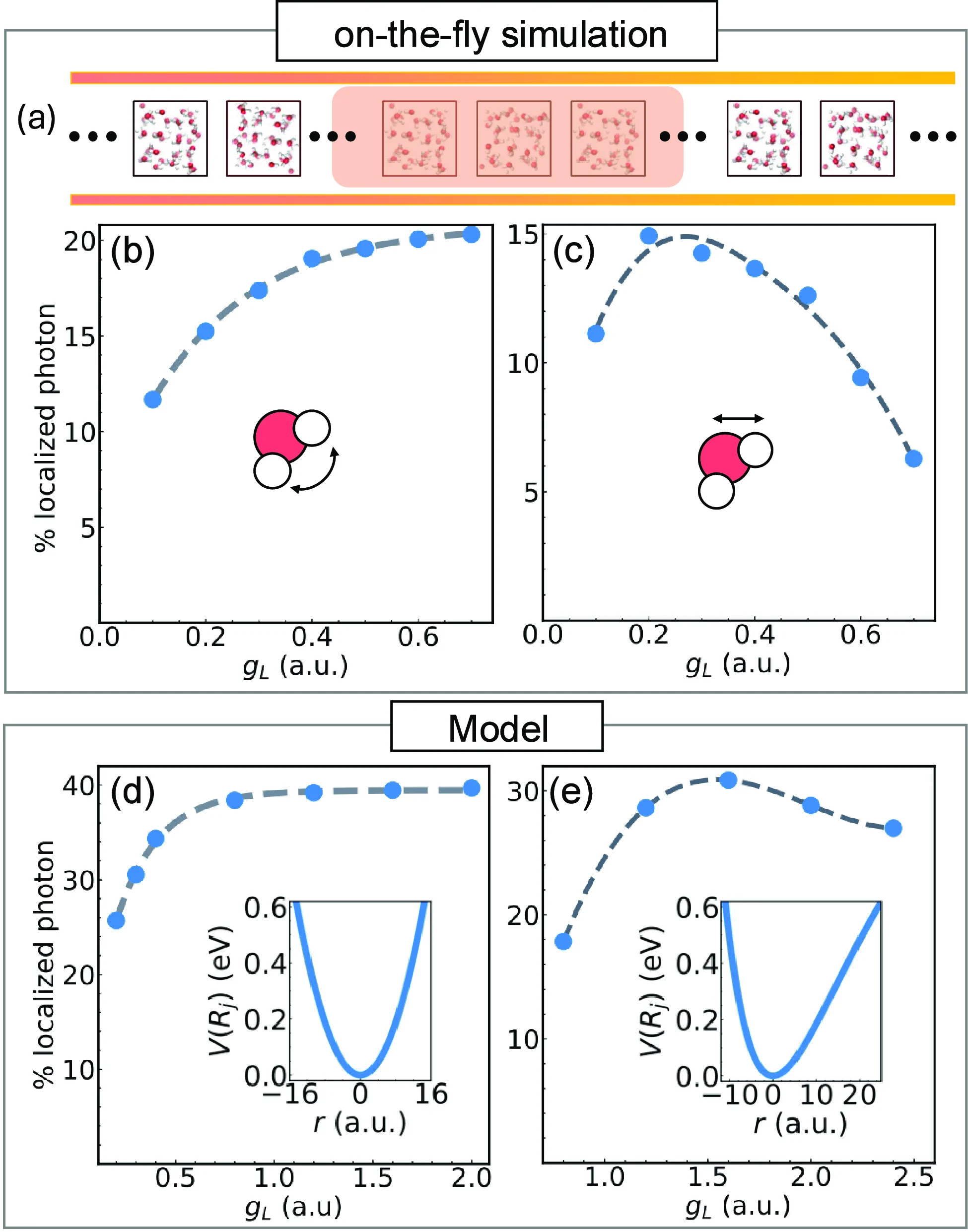
Capturing atomistic results using reduced models. (a) Selected window for computing % of localized photons is the center cell ± 50, spanning ∼10% of the total cavity length. (b) % of localized photons in simulation when ω_0_ = 0.16 eV at varying driving strengths *g*_L_ ∈ (0.00, 0.70) au. (c) % of localized photons in simulation when ω_0_ = 0.40 eV at varying driving strengths *g*_L_ ∈ (0.00, 0.70) au. (d) % of localized photons when using a simple harmonic oscillator model system. (e) % of localized photons when using a Morse potential model system.

Interestingly, the spatially resolved time-dependent photonic density is drastically altered while increasing the photonic driving strength *g*_L_ when setting ω_0_ = 0.40 eV, close to the anharmonic modes of water. Figure 2b2 and c2 demonstrates that the extent of the photonic localization is different at *g*_L_ = 0.3 and 0.6 au in that the localized portion disappears when using a relatively high photonic driving. This can be clearly observed in Figure 2d2, which displays the relative photonic density at 72 fs. Note the localized photonic density near the center of the cavity (*r* = 0) at *g*_L_ = 0.3 au (orange line) which is missing at *g*_L_ = 0.6 au (red line). These results also mirror the result obtained in many-body quantum dynamics of exciton–polaritons in the presence of exciton–exciton onsite interaction terms which can be viewed as anharmonicity.^27^ Note that molecular anharmonicity, modeled as a Morse potential, corresponds to an attractive many-body interaction term in the Bose–Hubbard picture. Due to this many-body attraction (which increases with increasing photon number), propagating photonic density is expected to pull away localized densities and suppress localization. Classically, a larger extent of driving is expected to dynamically tune the effective frequency of the anharmonic mode, which allows for the photonic population trapped near *k* ∼ 0 to scatter to higher *k* ≠ 0 modes with finite velocity, making this photonic density mobile. This mechanism is consistent with our observations.

To unambiguously demonstrate that the suppression of photonic localization originates from molecular anharmonicity, we compute photonic localization, $N_{\mathrm{loc}}$ , as a function of photonic driving and compare it with a reduced model system. We define photonic localization as the ratio of photons found in the middle of the cavity versus the entire cavity setup, expressed as

5 $$N_{\mathrm{loc}}=\frac{\int_{t-\Delta t}^{t+\Delta t}d\tau \int_{-r_{c}}^{+r_{c}}drN\left(r,\tau \right)}{\int_{t-\Delta t}^{t+\Delta t}d\tau \int_{-\infty }^{+\infty }drN\left(r,\tau \right)}$$

where *N*(*r*, τ) ≈ *∑*_*n*_ *N*_*n*_(τ)­δ­(*r* – *an*), *a* = 0.0427 μm is the lattice constant (which produces converged results; see Figure 1e), and *r*_c_ = 2.11 μm sets the bounds of the localized region (corresponding to ±50 unit cells) which is ∼10% of the total cavity length. In the following, we set *t* = 21 fs and use a finite Δ*t* = 6 fs to reduce noise. We note that this localization is unrelated to the recently observed nonlinear freezing of photonic density.^39^

Figure 3b presents the % photonic localization $\left(\%N_{\mathrm{loc}}\right)$ as a function of driving strength *g*_L_ when setting ω_0_ = 0.16 eV, close to the harmonic bending mode of water. At thermal equilibrium, under no driving, $\%N_{\mathrm{loc}}$ is expected to be ∼10%, consistent with the localized window occupying ∼10% of the total cavity length. This explains the $\%N_{\mathrm{loc}}$ as *g*_L_ → 0 in Figure 3b. As *g*_L_ is increased, the localized photonic density overcomes the thermal noise, and we consequently observe an increase in the % photonic localization in Figure 3b. Importantly, once *g*_L_ is sufficiently large (i.e., *g*_L_ > 0.4 au), the amount of photonic localization saturates.

The same is observed in Figure 3d when using a simple model system where the molecular degrees of freedom are described as a collection of harmonic oscillators. That is, we replace ${Ĥ}_{M_{n}}\to {Ĥ}_{M_{n}}^{\mathrm{mod}el}=\underset{j}{\sum }\frac{{p_{j,n}}^{2}}{2}+V\left(R_{j}\right)$ with $V\left(R_{j}\right)=\frac{1}{2}\Omega^{2}{R_{j}}^{2}$ , a harmonic potential (illustrated in the inset of Figure 3d), while we keep the rest of the terms identical in ${Ĥ}_{\mathrm{total}}$ . Further details can be found in the Supporting Information. As observed in Figure 3b, the photonic localization saturates at some *g*_L_.

Figure 3c presents the % photonic localization as a function of driving strength when setting ω_0_ = 0.40 eV, close to the anharmonic stretching modes of water. Importantly, we observe that the photonic localization nonlinearly depends on the photonic driving; it increases at smaller values of *g*_L_ (similar to Figure 3b) but then starts to decrease with increasing *g*_L_, indicating a suppression of localization as was discussed in Figure 2.

Figure 3e confirms that this nonlinear effect originates from molecular anharmonicity. Here, we consider a model molecular system where the molecular degrees of freedom are described as anharmonic oscillators, i.e., we set $V\left(R_{j}\right)=\frac{\Omega }{4\chi_{e}}{\left(1-e^{-\sqrt{2\Omega \chi_{e}}R_{j}}\right)}^{2}$ , a Morse potential (illustrated in the inset of Figure 3e), where χ_e_ = 0.08 au is the anharmonicity parameter (where χ_e_ → 0 means $V\left(R_{j}\right)\to \frac{1}{2}\Omega^{2}{R_{j}}^{2}$ ). The *g*_L_-dependent photonic localization presented in Figure 3e clearly reproduces the nonlinear, non-monotonic dependence of $N_{\mathrm{loc}}$ on *g*_L_, which was absent in Figure 3d. Overall, our results presented in Figure 2 and 3 clarify how molecular anharmonicity controls (multi)­photonic dynamics. We emphasize that these dynamical insights can only be captured when employing a mesoscale beyond long-wavelength description^35,37–39,48–54^ and that a single cavity mode limit adapted in recent theoretical works^30,32,55–63^ is not adequate.

## Mode-Selective Energy Transport

In Figure 4, we now focus on how molecular dynamics is also impacted due to light–matter coupling. Specifically, our results show that coupling to cavity photons enables mode-selective vibrational energy transport which can be tuned by altering the cavity mirror spacing (i.e., by changing ω_0_).

**Figure 4:**
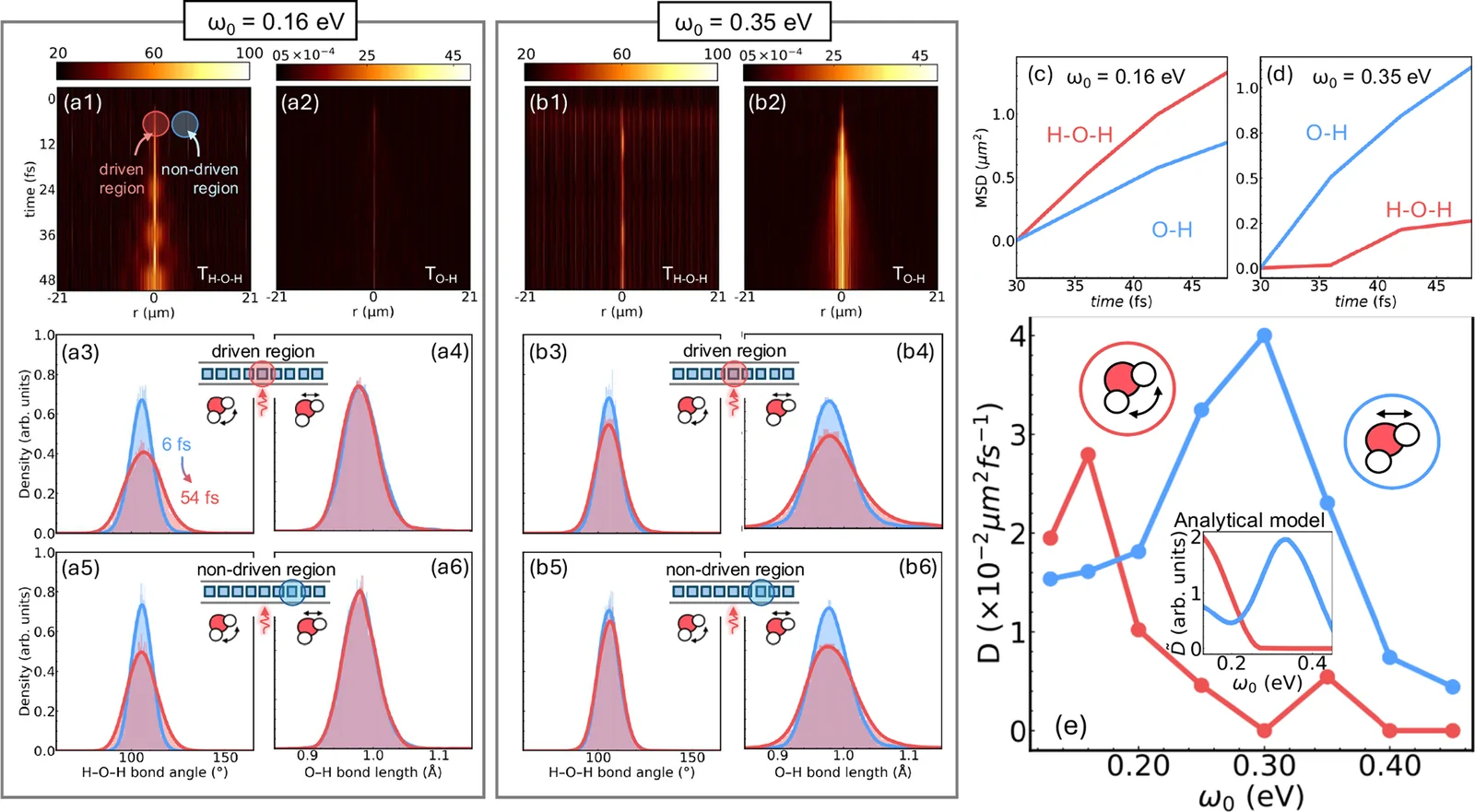
Mode-selective energy transport. (a) Cavity frequency ω_0_ = 0.16 eV, near bending mode. (a1) Effective temperature *T*_H–O–H_ diffusion over 54 fs. (a2) Effective temperature *T*_O–H_ diffusion over 54 fs. (a3 and a4) Distributions of (a3) bond angles and (a4) bond lengths in the driven region at *t* = 6 and 54 fs. (a5 and a6) Distributions of (a5) bond angles and (a6) bond lengths in the non-driven region at *t* = 6 and 54 fs. (b) Cavity frequency ω_0_ = 0.35 eV, near stretching modes. (b1) Effective temperature *T*_H–O–H_ diffusion over 54 fs. (b2) Effective temperature *T*_O–H_ diffusion over 54 fs. (b3 and b4) Distributions of (b3) bond angles and (b4) bond lengths in the driven region at *t* = 6 and 54 fs. (b5 and b6) Distributions of (b5) bond angles and (b6) bond lengths in the non-driven region at *t* = 6 and 54 fs. (c and d) MSDs of bending mode (red) and stretching mode (blue) energy diffusion at (c) ω_0_ = 0.16 eV and (d) ω_0_ = 0.35 eV. (e) Cavity frequency-dependent mode-resolved energy diffusion and comparison to analytical model.

Figure 4a and b presents the mode-selective vibrational energy transport at two different ω_0_. To quantify the vibrational energy transport, we compute the H–O–H bond angle and O–H bond length distributions. The corresponding probability densities (in arb. units) are presented in Figure 4a3–a6 and b3–b6. From these distributions, we compute a spatially resolved effective temperature (i.e., for each unit cell), defined as *T*_H–O–H_ = ⟨θ^2^⟩ – ⟨θ⟩^2^ and $T_{O-H}=\left\langle r_{O-H}^{2}\right\rangle -{\left\langle r_{O-H}\right\rangle }^{2}$ , where θ is the H–O–H bond angle and *r*_O–H_ is the O–H bond length. This particular choice of effective temperature follows from the fact that for perfectly harmonic potentials, such as $\frac{1}{2}\Omega^{2}R^{2}$ , the classical Boltzmann distribution leads to the relationship *T* ∝⟨*R* ^2^⟩ – ⟨*R*⟩^2^. Note that while the O–H stretching potential is anharmonic, the bond length variance still provides a convenient measure of spatial redistribution of the stretching excitation.

Figure 4a1 and a2 presents the spatially resolved time-dependent effective temperature when setting ω_0_ = 0.16 eV. The time-dependent *T*_H–O–H_ heatmap presented in Figure 4a1 shows that the unit cells progressively get “heated” (indicating energy transport) after a short-time excitation (which persists for the initial 10 fs) in the central region of the cavity with *r* ∈ [−0.234 μm, 0.234 μm]. In contrast, we observe significantly slower transport in Figure 4a2 which presents the time-dependent *T*_O–H_ heatmap.

A microscopic view is presented in Figure 4a3–a6. Figure 4a3 presents the density of the bending angle ρ_θ_ within the driven unit cells with *r* ∈ [−0.234 μm, 0.234 μm]. Clearly, ρ_θ_ is broadened over time, indicating the deposition of energy into the bending mode of water. Note that the bending mode is not directly coupled to the external laser, but it acquires energy indirectly from the cavity photon modes which are directly driven. Within the same time window, we observe little broadening of ρ_O–H_ (density of the O–H bond length), presented in Figure 4a4. Obviously, at much longer times, ρ_O–H_ will also broaden due to intramolecular IVR. Meanwhile, in Figure 4a5, we present the density of the bending angle ρ_θ_ within the non-driven unit cells (neighboring the driven unit cells), with *r* ∈ [0.255 μm, 0.468 μm]. We observe that ρ_θ_ is broadened over time, indicating cavity-enabled energy transfer. The same is not true in Figure 4a6 which shows marginal changes in ρ_O–H_.

When setting ω_0_ = 0.35 eV, which is closer to the stretching modes, the results presented in Figure 4b1–b6 show the opposite trend compared to Figure 4a1–a6. Here, the propagation of *T*_O–H_ (Figure 4b2) is more substantial than the propagation of *T*_H–O–H_ (Figure 4b1). This can also be observed by analyzing the microscopic picture presented in Figure 4b3–b6. Specifically, we see that both the driven and non-driven regions show a substantial broadening of ρ_O–H_ (compare Figure 4b4 and b6). In contrast, while the driven region displays a substantial broadening of ρ_θ_ (Figure 4b3), which is mediated by IVR, the broadening of ρ_θ_ in the non-driven region (Figure 4b5) is much less so, suggesting a much slower propagation of *T*_H–O–H_.

In Figure 4c and d, we present the mean square displacement (MSD) of *T*_H–O–H_ and *T*_O–H_. The MSDs are calculated as

6 $$\mathrm{MSD}\left(t\right)=\frac{\int_{-\infty }^{\infty }drr^{2}T_{j}\left(r,t+t_{s}\right)}{\int_{-\infty }^{\infty }drT_{j}\left(r,t+t_{s}\right)}-\frac{\int_{-\infty }^{\infty }drr^{2}T_{j}\left(r,t_{s}\right)}{\int_{-\infty }^{\infty }drT_{j}\left(r,t_{s}\right)}$$

where *T*_*j*_ ∈ [*T*_H–O–H_, *T*_O–H_], and *t*_s_ = 30 fs is a reference time. The MSDs for *T*_H–O–H_ and *T*_O–H_ at ω_0_ = 0.16 eV are presented in Figure 4c. It is clear that in the present scenario, the MSD for *T*_H–O–H_ is larger than for *T*_O–H_. In contrast, the MSDs presented in Figure 4d show the opposite behavior, *T*_O–H_ propagates much faster compared to *T*_H–O–H_ at ω_0_ = 0.35 eV, corroborating our findings in Figure 4a and b.

Finally, to illustrate the frequency-dependent and mode-selective nature of vibrational energy transport, quantified by *T*_O–H_ and *T*_H–O–H_, we compute the (effective) diffusion constant from our MSDs as $D=\frac{\mathrm{MSD}\left(t_{f}\right)}{2t_{f}}$ , where we set *t*_f_ = 18 fs (corresponding to *t* = *t*_f_ + *t*_s_ = 48 fs). Note that the MSDs presented here are not perfectly linear; nevertheless, this *D* allows for comparing the relative propagation of the effective temperatures. Our mesoscale simulations show that the diffusion of *T*_O–H_ and *T*_H–O–H_ can be tuned by changing ω_0_. For low ω_0_ (<0.2 eV), we find the diffusion of *T*_O–H_ to be much smaller than that of *T*_H–O–H_, which switches at ω_0_ ∼ 0.2 eV so that the diffusion constant for *T*_O–H_ becomes much larger than the diffusion constant for *T*_H–O–H_. At much larger ω_0_ (∼0.45 eV), both diffusion constants become negligible.

This cavity frequency dependence of mode-selective ‘energy’ diffusion can be rationalized using a crude analytical picture. Quantum mechanically, using Markovian Redfield dynamics, the diffusion constant is written as^64–66^

7 $$D_{\mathrm{QM}}\approx \underset{k}{\sum }\frac{{v_{k}}^{2}}{\Gamma_{k}}\rho_{kk}$$

where ρ_*kk*_ is the population of the *k*th state (where *k* is the wavevector), *v*_*k*_ = dω_*k*_/d*k* is the group velocity, and Γ_*k*_ is a scattering rate. When assuming a *k*-independent scattering rate, the diffusion constant is then proportional to the weighted average of the group velocity squared (i.e., $D_{\mathrm{QM}}\propto \underset{k}{\sum }{v_{k}}^{2}\rho_{kk}$ ). Noting that the number of photons at *k* is expected to be proportional to $|E\left(\omega_{k}\right){|}^{2}$ (where E(ω) is the sine-transformed E(t) in eq 3) and that the transition rate from photon to molecular vibrations is proportional to the oscillator strength *I*, we arrive at the proportionality $\rho_{kk}\propto I|E\left(\omega_{k}\right){|}^{2}$ . Following this, we arrive at a crude analytical expression

8 $$\mathrm{D̃}\left(\omega \right)=\frac{I}{\Delta \omega }\int_{\omega -\Delta \omega /2}^{\omega +\Delta \omega /2}d\omega {v_{k}}^{2}\left(\omega \right)|E\left(\omega \right){|}^{2}$$

where Δω = 50 meV accounts for a finite energy window which is populated, and we treat *I* as a free parameter (set to 1 for the H–O–H bending mode and 2.2 for the O–H stretching mode) which simply scales the overall line shape. Here, D̃(ω) should be considered proportional to the observed diffusion constant. We present $\mathrm{D̃}\left(\omega_{\theta }\right)$ and $\mathrm{D̃}\left(\omega_{v_{1}}\right)$ as functions of ω_0_ in the inset of Figure 4e. Despite the crude nature of this analytical expression, we find that our analytical model qualitatively reproduces the simulated ω_0_-dependent diffusion constants. This analysis reveals that energy transport associated with a particular molecular mode can be achieved on subpicosecond time scales by bringing the photonic dispersion close to resonance with that mode. This enables rapid, cavity-mediated through-space energy transport over propagation lengths reaching the micrometer scale.

In this work, using our mesoscale on-the-fly atomistic simulations, we provide mechanistic principles for cavity-enabled energy transport under VSC which extend to the experimental scale. Our results highlight the importance of going beyond a simple model molecular description of the matter subsystem and the need for adapting a beyond long-wavelength description of light–matter interactions to understand cavity-enabled dynamical phenomena.

We show that photonic transport is sensitive to the coupled molecular mode and its anharmonicity. We find that highly anharmonic molecular modes suppress localization of photonic density, especially at higher photonic driving. In contrast, the localization of photonic density becomes nearly independent of photonic driving when coupling to a harmonic molecular mode. We demonstrate this in cavity-coupled water, where the bending mode is harmonic (or weakly anharmonic), but the stretching modes are relatively anharmonic. We use reduced models to verify this mechanistic principle. Finally, we show that photon frequency at normal incidence can be used to tune the relative diffusion of mode-selective propagation of effective temperature (consequently energy transport), quantified here by the spatially resolved time-dependent variances of the bond angle and bond length distributions of water. We show that this mode selectivity can be understood as a consequence of the interplay between photonic group velocity and frequency-dependent laser intensity.

Overall, our work sheds light on the complex light–matter dynamics under VSC, paving the way for investigating how mode-selective transport may lead to mode-selective chemical dynamics.

## Supplementary Material



## Notes

Convergence with number of trajectories and details of the light–matter model system (PDF)
