# Supplementary material for: Mode-Selective and Anharmonicity-Dependent Energy Transport in Cavity-Coupled Water
Source: Nano Lett. 2026 Oct 9;26(41):14445–53. doi: 10.1021/acs.nanolett.6c04125 (PMC13693502; doi:10.1021/acs.nanolett.6c04125)
Supplement: Supplementary file 1 [file nl6c04125_si_001.pdf]

Mode-Selective and Anharmonicity-Dependent Energy Transport in Cavity-Coupled Water

Sachith Wickramasinghe,[†] Michael Fowler,[†] Gerrit Groenhof,[‡] and Arkajit Mandal[*,†]

†*Department of Chemistry, Texas A&M University, College Station, Texas 77843, USA*

‡*Department of Chemistry, University of Jyväskylä, 40014 Jyväskylä, Finland*

E-mail: mandal@tamu.edu

Convergence with Number of Trajectories

To support the validity of our calculations, we examine the convergence of the percentage of localized photons with respect to the number of independent trajectories. In Fig. S1, we present four individual trajectories and their percentage of localized photons (computed with Eq. 5 in the main text) for a driving strength of $g_L = 0.6$ a.u. In Fig. S1a, we observe no significant changes when more trajectories are averaged, meaning that we can achieve the ensemble-average behavior even with a single trajectory due to the mesoscale nature of the system. We show the photonic propagation over time in Fig. S1b-e when 1, 2, 3, and 4 trajectories are averaged, respectively.

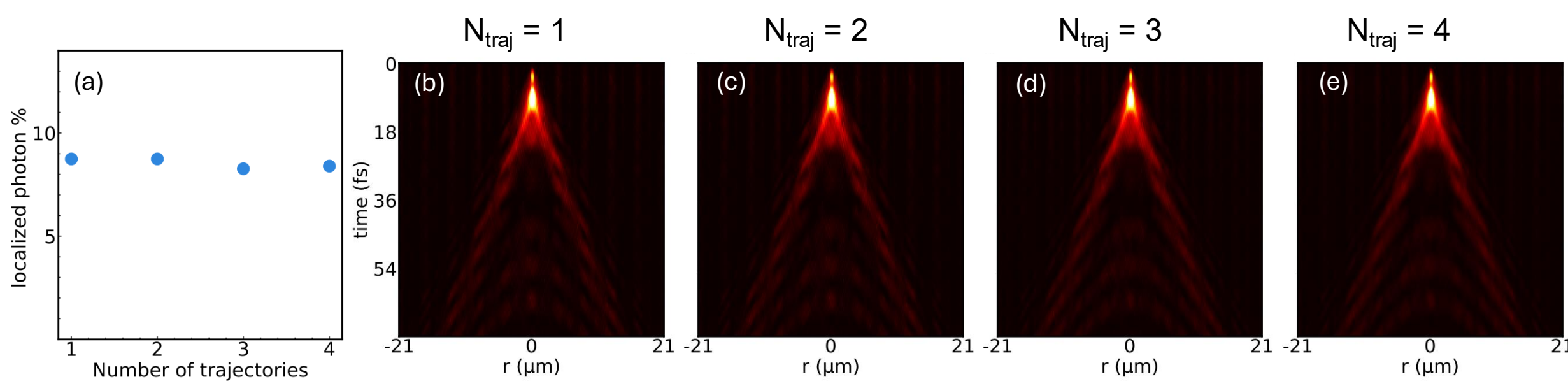

Figure S1: **Convergence with Respect to the Number of Trajectories.** (a) Percentage of localized photons convergence with number of trajectories. (b-e) Polaritonic propagation over time with respect to the number of individual trajectories averaged. We set $g_L = 0.6$ a.u. and $\omega_0 = 0.40$ eV.

Light–Matter Model System

To understand the anharmonicity-controlled transport observed in our simulations, we use the following reduced light-matter Hamiltonian for our model calculations:

$$H_{\mathrm{LM}} = H_{\mathrm{M}} + \frac{1}{2}\sum_k \left(p_k^2 + \omega_k^2 q_k^2\right) + \sum_n q_n \mu_n + \frac{1}{2\omega_0^2}\sum_n \mu_n^2. \tag{S1}$$

H_{M} represents the molecular Hamiltonian, which is written as a sum of N non-interacting

unit cells such that

$$H_{\mathrm{M}} = \sum_n H_{\mathrm{M}_n} = \sum_{n,j} T_{n,j} + V_n(\{R_{n,j}\}), \tag{S2}$$

where $T_{n,j}$ is the kinetic energy of the jth oscillator in the nth unit cell, and the corresponding anharmonic potential energy is

$$V_n(R_j) = \frac{\Omega}{4\chi_e}\left(1 - e^{-\sqrt{2\Omega\chi_e}\cdot R_{n,j}}\right)^2, \tag{S3}$$

where $\Omega = 0.43$ eV and $\chi_e = 0.08$ a.u. In Eq. S1, q_k and p_k are the reciprocal-space photonic position and momentum corresponding to the kth cavity mode with frequency ω_k, q_n is the real-space photonic position, and ω_0 is the cavity photon frequency at normal incidence. Here, μ_n is defined as

$$\mu_n = \sum_j R_{n,j} C_j, \tag{S4}$$

where $R_{n,j}$ is the position of the jth oscillator in the nth unit cell, and C_j is the corresponding coupling coefficient.

In an isotropic orientation, the molecular dipoles are uniformly oriented, and the probability distribution of the angle θ between a molecular dipole and the cavity polarization direction is given by

$$P_\mu(\theta) = \frac{1}{2}\sin(\theta), \qquad 0 < \theta < \pi. \tag{S5}$$

The projection of the dipole moment along the cavity polarization direction is proportional to $\cos(\theta)$. For an isotropic ensemble, this is uniformly distributed over [-1,1] and

symmetric around zero. We present this continuous distribution using a discrete set of N_m equally spaced points between [-1,1], obtained via midpoint discretization, such that, we define the coupling coefficient C_j as

$$C_j = \eta_b \big(1 - \frac{2j-1}{N_m}\big), \qquad j = 1, ..., N_m. \tag{S6}$$

We set $\eta_b = 0.000008$ a.u., and the number of oscillators ($N_m = 33$) was chosen to match the number of water molecules. Note that C_j is not n-dependent.
